# Molecular chaperone function of three small heat-shock proteins from a model probiotic species

**DOI:** 10.1007/s12192-022-01309-6

**Published:** 2022-11-22

**Authors:** Maria Teresa Rocchetti, Tiffany Bellanger, Maria Incoronata Trecca, Stephanie Weidmann, Rosella Scrima, Giuseppe Spano, Pasquale Russo, Vittorio Capozzi, Daniela Fiocco

**Affiliations:** 1 Department of Clinical and Experimental Medicine, University of Foggia, Via Pinto 1, 71122 Foggia, Italy; 2 Univ. Bourgogne, Franche-Comté, AgroSup Dijon, PAM UMR A 02.102, Dijon, France; 3 Department of Agriculture Food Natural Science Engineering (DAFNE), University of Foggia, Via Napoli 25, 71122 Foggia, Italy; 4 Department of Food, Environmental and Nutritional Sciences, University of Milan, Via Luigi Mangiagalli 25, 20133 Milano, Italy; 5 Institute of Sciences of Food Production, National Research Council (CNR) of Italy, C/O CS-DAT, Via Michele Protano, 71122 Foggia, Italy

**Keywords:** Protein aggregation, Heat stress, Holdase, Lipochaperone, Membrane fluidity, sHSP

## Abstract

Small heat-shock proteins (sHSP) are ubiquitous ATP-independent chaperones that prevent irreversible aggregation of heat-damaged denaturing proteins. *Lactiplantibacillus plantarum* is a widespread Gram-positive bacterium with probiotic claims and vast potential for agro-food, biotechnological and biomedical applications. *L. plantarum* possesses a family of three sHSP, which were previously demonstrated to be involved in its stress tolerance mechanisms. Here, the three *L. plantarum* sHSP were heterologously expressed, purified and shown to have a chaperone activity in vitro, measuring their capacity to suppress protein aggregation, as assayed spectrophotometrically by light scattering. Their anti-aggregative capacity was found to be differently influenced by pH. Differences were also found relative to their holdase function and their capacity to modulate liposome membrane fluidity, suggesting interplays between them and indicating diversified activities. This is the first study assessing the chaperone action of sHSP from a probiotic model. The different roles of the three sHSP can increase *L. plantarum*’s capabilities to survive the various types of stress characterising the diverse habitats of this highly adaptable species. Reported evidence supports the interest in *L. plantarum* as one of the model species for bacteria that have three different sHSP-encoding genes in their genomes.

**Supplementary Information:**

The online version contains supplementary material available at 10.1007/s12192-022-01309-6.

## Introduction

Small heat-shock proteins (sHSP) are ATP-independent molecular chaperones found in all life kingdoms (Haslbeck et al. 2019). Their structure is characterised by a low molecular weight (12–43 kDa) and the presence of a central, highly conserved α-crystallin domain (approximatively 100 amino acids long), flanked by N- and C-terminal regions with more variable sequence and length. sHSP bind denaturing proteins, thereby preventing their irreversible aggregation and maintaining them in a native-like refolding-competent state, so that, upon release, sHSP-trapped substrates can recover their native conformation thanks to the ensuing recruitment of ATP-dependent chaperones (Carra et al. 2019; Obuchowski et al. 2021; Reinle et al. 2022).

sHSP are key players in the molecular stress response: they protect cells from damage under adverse conditions, mainly thermal stress, but even support protein homeostasis under physiological conditions (Haslbeck and Vierling 2015). The chaperone properties of sHSP seem closely connected to their capacity to form oligomeric quaternary structures of different sizes that encage the unfolding substrate, shielding it from the external environment and protecting it from further aggregation/precipitation (Maitre et al. 2012, 2014; Fleckenstein et al. 2015; Żwirowski et al. 2017). This preservation of misfolded proteins from uncontrolled aggregation is described as holdase function and can be measured by assessing the prevention of insoluble protein agglomerations (Mogk et al. 2019; Reinle et al. 2022).

Although the main substrates of sHSP are cytoplasmic proteins, some microbial sHSP were also found to associate with membrane lipids, thereby modulating the bilayer fluidity (Sales et al. 2000; Török et al. 2001) through a lipochaperone function. For instance, in the wine-associated lactic acid bacterium *Oenococcus oeni*, a membrane-stabilising role was ascribed to the sHSP Lo18 (Maitre et al. 2014; Darsonval et al. 2016).

*Lactiplantibacillus plantarum* is a versatile species of lactic acid bacteria (LAB) that inhabits vegetables and human-associated niches, including fermented food, gut and vaginal mucosa (Siezen et al. 2010). *L. plantarum* strains exhibit probiotic attributes and hold great potentialities for agro-food, biotechnological and biomedical applications (Seddik et al. 2017; Rocchetti et al. 2021; Zhang et al. 2022). In particular, *L. plantarum* strain WCFS1 has been thoroughly studied and is considered a model for gaining insights into interactions between probiotic microorganisms and human hosts (van den Nieuwboer et al. 2016). *L. plantarum* WCFS1 genome, one of the largest among lactobacilli, harbours three diverse sHSP-encoding genes, referred to as *hsp1*, *hsp2* and *hsp3* (Kleerebezem et al. 2003; Siezen et al. 2012). In effect, bacterial genomes usually contain only one or two sHSP encoding genes; however, in about 10%, there are three sHSP genes, hence being a minority but well-represented condition, particularly prevalent in non-pathogenic species (Kriehuber et al. 2010; Haslbeck 2016).

In earlier studies, we found that the three *hsp* genes of *L. plantarum* are differently organised and transcriptionally regulated (Spano et al. 2004, 2005; Fiocco et al. 2010; Russo et al. 2012; Bove et al. 2013). Moreover, analysis of the knock-out (KO) *L. plantarum* mutants for each of the *hsp* genes suggested their diversified contribution to stress response, with HSP2 owing a housekeeping function for proteome homeostasis and possibly controlling membrane fluidity (Capozzi et al. 2011b, a), HSP3 involved in thermotolerance, HSP1 playing cryoprotective functions (Arena et al. 2019) and potentially involved in probiotic (immunomodulatory) interactions with the host (Longo et al. 2021).

In this study, we report the biochemical characterisation of *L. plantarum* WCFS1 sHSP: by cloning and heterologous expression of the three *hsp* genes, the recombinant, native forms of HSP1, HPS2 and HSP3 were obtained and assayed in vitro for molecular chaperone activity and for the ability to modulate membrane fluidity.

## Material and methods

### Bacterial strains and culture conditions

The bacterial strains used in this work are listed in supplementary Table S1. *Escherichia coli* strain MACH-1-T1 was used for DNA cloning, while *E. coli* strain BL21 (DE3) was used for the expression of recombinant proteins (recombinant HSPs expression through the SUMO fusion technology). *E. coli* was grown in Luria–Bertani (LB) broth at 37 °C, with shaking. Kanamycin (50 µg mL^−1^) was added when required. *L. plantarum* WCFS1 was grown in De Man Rogosa Sharpe (MRS, Liofilchem, Italy) broth (pH 6.2), at 30 °C, without shaking. Agar (15 g L^−1^) was added to obtain solid media.

### DNA extraction

Plasmids were isolated from *E. coli* using QIAprep spin miniprep kits (Qiagen, Hilden, Germany). *L. plantarum* genomic DNA was isolated using a microbial DNA extraction kit (Cabru, Milan, Italy), according to the manufacturer’s guidelines.

### Cloning, expression and purification of recombinant sHSP

The open reading frames of the genes encoding small heat-shock proteins, i.e., *hsp1*, *hsp2* and *hsp3* (locus tag lp_0129, lp_2668 and lp_3352, respectively, on the *L. plantarum* WCFS1 complete genome, NCBI BioProject: PRJNA356) were amplified from the chromosomal DNA of *L. plantarum* WCFS1 using Platinum Taq DNA Polymerase High Fidelity (Life Technologies, Carlsbad, CA, USA), specific forward and reverse primers and the following thermal profile: 2 min at 94 °C, 35 cycles comprising 15 s at 94 °C, 30 s at 55 °C and 50 s at 68 °C, and 7 min at 68 °C. All the plasmids and oligonucleotides used in this work are listed in Table S1.

After checking specific amplification and correct amplicon size by agarose gel electrophoresis, the PCR products were T/A cloned into pET SUMO vector, using the pET SUMO Protein Expression System kit (Invitrogen, Life Technologies), according to the manufacturer’s instructions, to generate pET-SUMO-HSP1, pET-SUMO-HSP2 and pET-SUMO-HSP3, respectively, which encode HSP1, HSP2 or HSP3 with an extra N-terminal polyhistidine (6xHis) tag and a SUMO fusion protein. Ligations were transformed into Mach1-T1^R^ chemically competent *E. coli* cells. Transformed *E. coli* colonies containing the recombinant vectors with the correct orientation were screened and selected by PCR (using T7 promoter FOR and HSP REV primers). Plasmids were purified using Qiaprep Spin Miniprep kit (Qiagen, Valencia, CA, USA) and sequenced using the Big-dye Terminator kit (Thermo Fisher Scientific, Waltham, MA, USA), with primers SUMO Forward and T7 reverse (both from Invitrogen), to check for correct cloning and absence of any mutation in the *hsp* open reading frames (ORF). Plasmids were then transformed into chemically competent *E. coli* BL21 (DE3) cells, and the expression of the corresponding 6xHis-tagged-SUMO-HSP fusion proteins was induced by adding 1 mM isopropyl-β-D-Thiogalactoside (IPTG) to exponentially growing cultures of *E. coli* in LB medium containing 50 μg mL^−1^ of kanamycin. The expression of recombinant fusion 6xHis-SUMO-HSP proteins was checked through sodium-dodecyl-sulphate–polyacrylamide gel electrophoresis (SDS-PAGE). The recombinant fusion sHSP were purified by affinity chromatography, under native conditions, using the Ni–NTA agarose purification system (Novex, Life Technologies), following the manufacturer’s instructions. After dialysis, 6xHis-tagged recombinant fusion sHSP were digested with SUMO-protease (Invitrogen by Life Technologies), in order to remove the N-terminal peptide containing the 6xHis- and SUMO-tag, therefore generating native sHSP (i.e., without any additional amino acids between the SUMO protease cleavage site and the start of sHSP). Undigested recombinant fusion sHSP, 6xHis-SUMO-tag peptides and SUMO protease were removed from the cleavage reaction by affinity chromatography on HisPur™ Ni–NTA Resin (Thermo Fisher Scientific) by batch method. Purified native sHSP were dialysed against 50 mM sodium phosphate (NaP) buffer at pH 7.0, concentrated, spectrophotometrically quantified and stored in aliquots at − 30 °C. Purification of sHSP was checked by SDS-PAGE, using 4–15% MP TGX Stain-Free precast polyacrylamide gel (Bio-Rad Laboratories, Hercules, CA, USA), which was further stained with colloidal Coomassie G-250 (Bio-Rad Laboratories) in order to check the absence of any additional bands besides that of sHSP. Purified sHSPs were analysed by matrix-assisted laser desorption ionization-time of flight-mass spectrometry (MALDI-TOF–MS, Autoflex III™, Bruker Daltonics, Bremen, Germany) to evaluate their molecular weight (see supplemental information). Some biochemical features of *L. plantarum* sHSP, along with their deduced aminoacid sequences, are reported as supplemental information (Table S2).

### Chaperone activity assay

The molecular chaperone activity of sHSP was evaluated by their ability to protect a model protein substrate, i.e., citrate synthase (CS), from thermal aggregation. According to Buchner and Grallert (1998), pig heart CS (from Sigma Aldrich, St. Louis, MO, USA) was dialysed with TE buffer (50 mM Tris–HCl, 2 mM EDTA, pH 8), then concentrated to 17 mg mL^−1^ at 4 °C and centrifuged at 14,000 × rpm, 30 min at 4 °C, to remove precipitated protein-complexes. The supernatant containing undenatured CS was concentrated to 30 µM (monomer) and stored in aliquots at − 20 °C.

Thermal aggregation of CS was monitored over time by measuring absorbance at 360 nm (*A*_360_), which reflects the formation of insoluble protein aggregates, as previously described (Buchner and Grallert 1998; Maitre et al. 2012). Briefly, CS (0.3 µM) was denatured at 45 °C in 50 mM sodium phosphate buffer at pH 5.5, 7.0 or 8.0 in the presence or absence of 0.3, 1.2, 4.8 or 9.6 µM of each sHSP, corresponding to molar ratios (sHSP:CS) of 1:1, 4:1, 16:1 and 32:1, respectively. Aggregation of CS was measured in a quartz cuvette by an Agilent Cary 3500 UV–Vis spectrophotometer equipped with thermostated cell holder and stirrer. The measurements were performed over a 20 min period, recording *A*_360_ at 0.1 s intervals. Experiments were performed in triplicate. Lysozyme (Sigma–Aldrich), which shares a similar molecular mass with sHSP, was used as a negative control protein, i.e., devoid of any known molecular chaperone activity.

### Holdase activity

Solubility determination was assessed as previously described (Fleckenstein et al. 2015). Briefly, 10 µg of CS in 50 mM sodium phosphate buffer pH 7.0 were incubated for 1 h at 45 °C in the presence or absence of HSP1, HSP2 or HSP3 or with a mixture of the three sHSP, at a molar ratio of 16:1 (sHSP:CS). Samples were centrifuged at 20,800 × g for 10 min at 4 °C to separate the soluble fraction from the pellet (i.e., containing aggregated proteins). Proteins in the soluble fraction were precipitated by cold acetone (10 min on ice) and centrifuged at 14,000 × rpm, 30 min at 5 °C. The precipitated proteins and the pellet were solubilised by the Laemmli buffer (Bio-rad) and resolved on a hand-casting 12% TGX Stain-Free polyacrylamide gel (Bio-rad), further stained with colloidal Coomassie G-250. Gel images were acquired by Gel Doc instrument (Bio-rad) and quantified by Image software. Ten µg of CS (0.3 μM in 50 mM sodium phosphate buffer pH 7.0), processed as all holdase assay samples, were used as control.

### Liposomes preparation and membrane fluidity measurements

Liposomes were prepared with total lipids from 30 absorbance units of *L. plantarum* cells harvested in exponential-phase (*OD*_600_ = 0.7–0.8) during growth in optimal culture conditions. Lipid extraction and liposome preparation were carried out as described by Maitre et al. (2012) with slight modifications. Briefly, after lipid extraction, according to Bligh and Dyer (1959), a film was obtained by evaporation of chloroform using a nitrogen flux. Then, a pre-warmed 50 mM phosphate buffer pH 7 at 55 °C was added, and the sample was gently mixed, sonicated twice for 2 min (Branson Ultrasonics™ CPX-952-138R, Branson Ultrasonics, Brookfield, CT, US) and incubated for 4 h at 55 °C. The lipid particles were then extruded through a polycarbonate membrane with 1 µm diameter pores to obtain the liposomes. Liposomes were stored at 4 °C during a 1-week maximum.

Fluorescence anisotropy, which is inversely proportional to membrane fluidity, was measured in a quartz cuvette filled with 250 µL of liposomes prepared as described above, containing 3 µM of 1,6-diphenyl-1,3,5-hexatriene (DPH) probe (Sigma Aldrich) and in the absence or the presence of 10 µM of purified HSP1, HSP2 or HSP3, using a mass ratio of 1:2 (m/m) between sHSP and liposomes. Lysozyme (10 µM) was used as a negative control. Measurements were performed for 30 min (1 determination every 10 s). After probe insertion (10 min at 10 °C), a linear gradient of temperature from 15 to 65 °C (increase of 2 °C per minute) controlled by a Peltier system (QNW TC1 temperature controller, Quantum Northwest, Liberty Lake, WA, USA) was applied to the liposome suspension. Anisotropy measurements were carried out using a Fluorolog 4 spectrofluorimeter (FLUOROLOG-4, Jobin Yvon Inc, USA), and anisotropy values were calculated according to Shinitzky and Barenholz (1978). Excitation and emission wavelengths were 360 nm and 431 nm, respectively. Each experiment was done in triplicate.

### Statistics

Quantitative variables were expressed as mean ± SD, unless otherwise indicated; their differences were tested by the nonparametric Mann–Whitney *U* test. *p* values < 0.05 were considered statistically significant. For all analyses, the Statview software package, SAS (v. 5.0), was used.

## Results

### Chaperone-like activity of sHSP

**Fig. 1:**
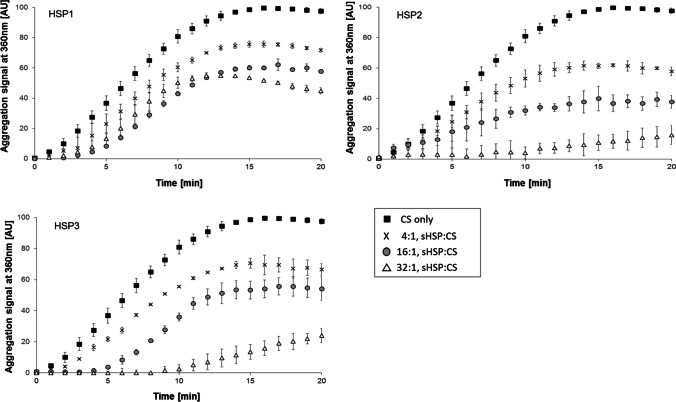
Dose-dependent inhibition of citrate synthase (CS) aggregation by *L. plantarum* sHSP. CS (0.3 µM) was incubated at 45 °C with increasing concentrations of HSP1, HSP2 or HSP3, and protein aggregation was monitored by the increase in light scattering at 360 nm. The saturation signal was normalised to 100. CS alone, solid square; CS + 1.2 µM sHSP (molar ratio 4:1, sHSP:CS), crosses; CS + 4.8 µM sHSP (molar ratio 16:1), grey circles; CS + 9.6 µM sHSP (molar ratio 32:1), open triangle. Mean and SD from at least 2 experiments

The disaggregase activity of sHSPs was evaluated in vitro by assessing their ability to protect a non-native model substrate (CS) from thermal aggregation. When heated at 45 °C, CS forms insoluble aggregates that scatter light at 360 nm (Fig. 1, solid squares). The addition of increasing amounts of purified HSP1, HSP2 or HSP3, at molar ratios of 4:1, 16:1 and 32:1 (sHSP:CS), caused a dose-dependent decline in light scattering, which can be attributed to the capacity of sHSP to prevent CS thermal-induced aggregation. In detail, upon 20 min heat exposure, at molar ratios 4:1, 16:1 and 32:1 (sHSP:CS), HSP1 caused a drop of the aggregation signal to approximatively 70%, 60% and 45%; HSP2 to about 60%, 30% and 15%; and HSP3 to approximately 65%, 55% and 25%, respectively, when compared to the control (CS only). The addition of each sHSP at a molar ratio of 1:1 relative to CS resulted in a light scattering signal mostly overlapping with that found at a 4:1 ratio (Fig. S1). The observed anti-aggregative effect was specific and depended on the presence of sHSP, as negative control solutions containing CS with a molar excess of nonchaperone control protein (i.e., lysozyme) did not result in attenuated aggregation; on the other hand, sHSP alone did not aggregate (Fig. S2).

In order to understand whether the three sHSP could cooperate to prevent thermal aggregation of proteins, aggregation assays were carried out incubating CS with a mixture of all three sHSP together, each at a concentration of 0.4 µM or 1.6 µM in order to get an overall sHSP concentration of 1.2 µM or 4.8 µM, corresponding to a molar ratio of 4:1 or 16:1 (sHSP:CS). The aggregation was inhibited but without any apparent synergistic effect (Fig. S3), as the extent of inhibition was similar to that observed for the single sHSP, when used at the same ratios (Fig. 1).

**Fig. 2:**
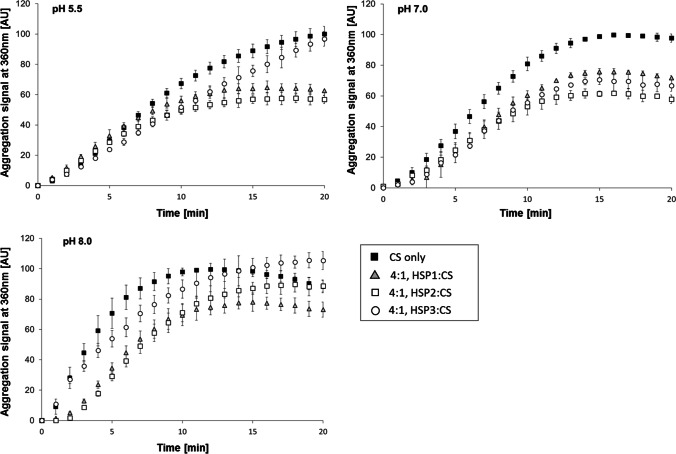
The effect of pH on the chaperone activity of HSP1, HSP2 and HSP3. Thermal induced aggregation of 0.3 µM citrate synthase (CS), solid squares, was monitored in 50 mM Na-phosphate buffer at pH 5.5, pH 7.0 and pH 8.0 in the presence of 1.2 µM HSP1 (grey triangle), or HSP2 (open square), or HSP3 (open circle), (4:1 molar ratio, sHSP:CS). The cuvette was incubated at 45 °C, and the protein aggregation was monitored by detecting light scattering at 360 nm. The saturation signal was normalised to 100. Mean and SD from at least 2 experiments

The chaperone activity of each sHSP was also examined as a function of the pH. Heat-induced aggregation assays of CS, incubated or not with a 4:1 molar excess of each sHSP, were carried out at pH values of 5.5 and 8.0 and compared with those performed at 7.0 (Fig. 2). At pH 7.0, the anti-aggregative effect of the three sHSP was similar, with HSP2 exerting a more evident anti-aggregative capacity. At acidic pH (5.5), both HSP1 and HSP2 retained activity (i.e., light scattering signal decreased by approximatively 50%), while HSP3 seemed to loose its aggregation-inhibiting properties; at pH 8.0, in the presence of either HSP1 or HSP2, CS aggregation was still decelerated compared to control, though, after almost 15 min, the light scattering signal in the presence of HSP2 tended to overlap with that of CS alone; conversely, the presence of HSP3 would not protect the substrate from aggregation.

Overall, while HSP1 and HSP2 seem to function, to a different extent, in a pH range spanning from 5.5 to 8.0, HSP3 exhibits a narrower pH interval for optimal anti-aggregative activity.

### Holdase function

sHSP should associate quite strongly with the unfolding substrates, holding them long enough before involving other members of the chaperone machinery. Hence, such sHSP holdase function should decrease the amount of precipitating substrate, while augmenting its soluble fraction.

**Fig. 3:**
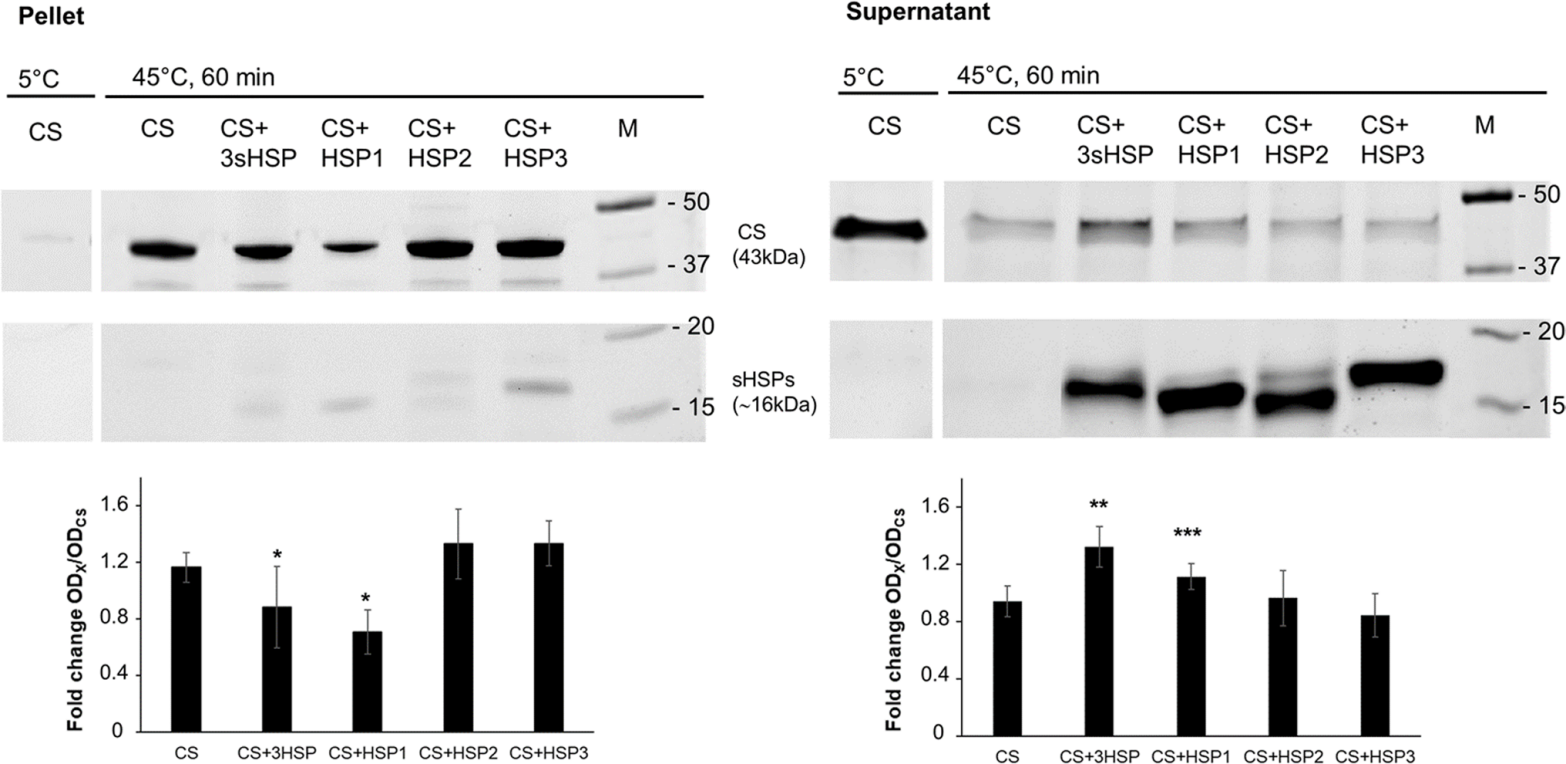
Holdase activity of *L. plantarum* sHSP. The solubility of a heat destabilised (45 °C, 60 min) CS in the absence and presence of HSP1, HSP2, HSP3 or all three sHSP (molar ratio sHSP: CS = 16:1) was determined by separation into supernatants and pellet fractions. Pellet (aggregated CS) was isolated from supernatants (soluble CS) by centrifugation, and both fractions were analysed by SDS-PAGE. A representative SDS-PAGE of the analysed fractions is reported. The same amount of CS not exposed to thermal stress (5 °C) represents the negative and positive control for pellet and supernatant, respectively. M, molecular weight markers. *p < 0.05 vs CS, CS + HSP2, CS + HSP3; **p < 0.05 vs CS, CS + HSP2, CS + HSP3; ***p < 0.05 vs CS + HSP3

In order to test the potential holdase activity of HSP1, HSP2 and HSP3, the solubility of heat-denatured model substrate CS was monitored in the presence and absence of each of the three HSP, using a molar ratio of 16:1 (sHSP:CS). Such ratio was found to markedly slow down aggregation (i.e., for all three sHSP, light scattering of CS declined by ≥ 40%, upon 20 min heat treatment, see above, Fig. 1), but not too much to prevent us from detecting potential differences between the sHSP in their holdase activity. With the aim to assess any cooperative effects between sHSP, the assay was also performed with a mixture of the three sHSP. After 1-h incubation at 45 °C, the amount of soluble (i.e., non-aggregated) and precipitated (i.e., aggregated) CS was determined by separation into supernatant and sediment fractions, respectively, via centrifugation and SDS-PAGE analysis (Fig. 3). Compared to control (i.e., CS alone exposed to 45 °C, lane 1, Pellet), when HSP1 was present, the pellet fraction contained a significantly decreased amount of precipitated CS; upon incubation with the mixture of three sHSP, a slightly but significantly lower level of precipitated CS was found. On the other hand, in the presence of either HSP2 or HSP3, the amount of precipitated CS was similar to that of control. Besides, low levels of each HSP could be detected in the pellet (Fig. 3, Pellet). Conversely, there was a significant increase of CS recovered in the soluble fraction when this substrate was incubated with the mixture of all three HSPs or, to a lower extent, with HSP1; whereas the amount of soluble CS was similar to heat-exposed control in presence of HSP2 or HSP3 (Fig. 3, Supernatant). Moreover, most of the sHSP were observed in the soluble fraction, indicating that these proteins, coherently with their putative function, do not appear to be aggregated by heat.

Taken together, these findings indicate that, under the tested conditions, HSP1 can bind aggregating substrates strongly enough thus reducing, though not suppressing completely, their precipitation, whereas HSP2 and HSP3, although capable of slowing down initial aggregation (Fig. 1), cannot maintain CS in a soluble form during prolonged heat exposure. Interestingly, co-incubation with a mixture of the three HSP resulted in the highest holdase activity (i.e., highest amount of CS in the soluble fraction), suggesting that they could act synergically in keeping the substrate in solution.

### Lipochaperone activity

**Fig. 4:**
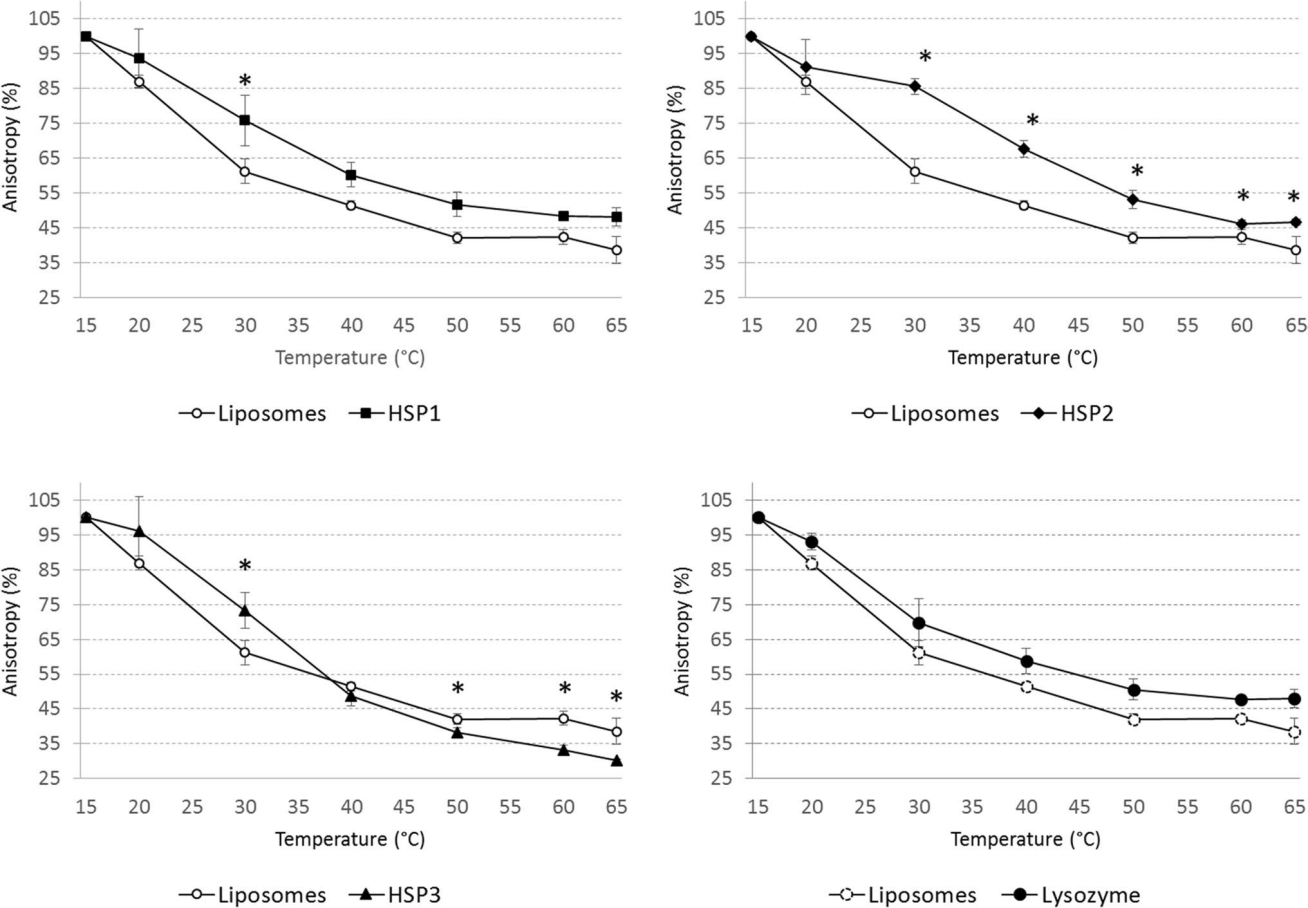
The effect of HSP1, HSP2 or HSP3 on membrane fluidity of lipid vesicles derived from *L. plantarum*. Fluorescence anisotropy of DPH probe inserted into vesicles was measured according to the temperature increase, in the absence (open circle) or presence of HSP1, HSP2 or HSP3 (solid symbols). Anisotropy was also measured in presence of lysozyme (solid circle), as a negative control. Mean and SD from at least 3 independent experiments. *p < 0.05, Mann–Whitney *U* test

Liposomes formed by lipids extracted from *L. plantarum* cells, collected during exponential growth, were mixed with DPH probe. Then, the variation of membrane fluidity was measured by steady-state fluorescence anisotropy of DPH during temperature ramping (15 °C to 65 °C), in the absence or presence of HSP1, HSP2 or HSP3 (Fig. 4). The gradual decrease of the anisotropy values reflected the increasing fluidisation of liposomes. Increasing the temperature up to 65 °C caused the liposomes to become more fluid, reaching 38.6% of the initial anisotropy value; similar results were obtained in presence of lysozyme, which was used as a negative control. The addition of HSP1 did not seem to have any impact on the regulation of membrane fluidity, except at low temperatures of 30 °C. A significant lipochaperone activity was observed for HSP2, which allowed to limit the fluidification of liposomes from 30 °C. At this temperature, the observed anisotropy was 85.5% of the initial value, compared to 61.2% in the absence of HSP2 (*p* < 0.05). The rigidifying effect of HSP2 continued throughout the thermal ramp allowing to reach 46.5% of initial anisotropy at 65 °C (Fig. 4). The anisotropy profile obtained for HSP3 was quite different. A limitation of membrane fluidisation was observed at 30 °C (*p* < 0.05), but this effect did not persist during the thermal ramp, as the liposomes become more fluid from 50 °C.

## Discussion

sHSP are molecular chaperones that associate with misfolded and/or unfolding proteins, preventing their uncontrolled aggregation and maintaining them in a condition that facilitates subsequent refolding or degradation by other members of the cell chaperone systems. *L. plantarum* is a widespread member of LAB, found in habitats where it is exposed to several kinds of stress. Unlike the majority of lactobacilli, possessing single sHSP, *L. plantarum* owns three different members of this chaperone family (Capozzi et al. 2011b). Such redundancy may afford a flexible response to variable environmental conditions, thus contributing to the high adaptability of this species (Han et al. 2008; Papadimitriou et al. 2016). Indeed, the presence of three HSP, which may have specialised for diverse functions or stress conditions, can contribute to the robustness of probiotics and biotechnologically relevant microbial species, which, for their application, undergo severe and diversified stress (Fiocco et al. 2020).

Although bacterial sHSP have been thoroughly studied (Obuchowski and Liberek 2020; Obuchowski et al. 2021), chiefly in model species such as *E. coli* (Piróg et al. 2021), only a very few works have investigated the sHSP of probiotic microorganisms (Capozzi et al. 2011b; Khaskheli et al. 2015). The involvement of *L. plantarum* sHSP in stress response strategies was demonstrated previously, mainly by expression, gene knockout (KO) and phenotypic studies (Fiocco et al. 2007; Capozzi et al. 2011a; Arena et al. 2019). In this work, by monitoring spectrophotometrically the turbidity of heat-destabilised protein solutions, we prove that all three sHSP have a chaperone role in vitro, as they could delay the thermal aggregation of a non-native substrate. Indeed, though assays based on light-scattering signal measurements are rather qualitative, they are specific for molecular chaperone activity (Buchner and Grallert 1998). The observed anti-aggregative activity was sHSP dose-dependent, thus pointing to a specific effect. In this regard, we found that, compared to HSP1, HSP2 and HSP3 were more effective in delaying aggregation. Indeed, over a 20-min heat exposure, the aggregation signal was reduced to 15% and 25% when CS was incubated with 32:1 molar excess of HSP2 and HSP3, respectively. Aggregation was highly inhibited only when *L. plantarum* sHSP concentration far exceeded that of the unfolding substrate (i.e., molar ratio of 16:1 and 32:1, sHSP:CS). This might depend on an intrinsically low activity of the purified recombinant sHSP or on the conditions used for the assays. However, it may even reflect that sHSP action relies on the formation of large assemblies that capture the denaturing proteins and physically isolate them from the external environment (Reinle et al. 2022). Therefore, numerous sHSP monomers would be required for efficient substrate trapping, and, similarly to other known sHSP, *L. plantarum* HSP2 and HSP3 could work as aggregation-preventing chaperones when present in excess. Besides, though all three HSP were previously found to be induced by heat (Spano et al. 2004, 2005; Russo et al. 2012), earlier studies indicated also that HSP1 might protect cell from freeze–thaw damage (Arena et al. 2019); therefore, its chaperone activity might have specialised rather for cold-related stress.

A pH-dependent chaperone activity has been observed earlier for sHSP, from both microbes (Maitre et al. 2012) and animals (Chernik et al. 2004; Fleckenstein et al. 2015), as pH is known to modulate their quaternary structures. Here, *L. plantarum* sHSP were found to respond differently to pH. While all three sHSP seemed to function at neutral pH (pH 7.0), only HSP1 and HSP2 were active at pH 5.5, i.e., a value which is observed in the cytoplasm of acid tolerant lactobacilli, including *L. plantarum*, when living in acidic environment (McDonald et al. 1990; Siegumfeldt et al. 2000). Conversely, at pH 8.0, which is a value that can be reached in the cytoplasm of alkali-resistant *L. plantarum* (Sawatari and Yokota 2007), only HSP1 retained a significant anti-aggregative ability. Such diversified pH effect might underlie the protection of cellular proteins under various conditions, thus enabling the growth of *L. plantarum* over a wide pH range and confirming the broad stress tolerance of this species (Parente et al. 2010). Interestingly, previous studies demonstrated that the transcription of *hsp1* and, to a lower extent, *hsp2* was strongly induced in the acidic sectors of a system simulating the gastro-intestinal transit (Bove et al. 2013). Besides, at low pH, the growth of *L. plantarum hsp1* KO mutant strain was much slower than the *hsp3* mutant (Arena et al. 2019). Thus, our present data would corroborate further the involvement of HSP1 and HSP2 in coping with acid stress, highlighting a differentiation in the role of the three sHSP in stress response.

sHSP work within a protein quality control network comprising holdases, foldases, unfoldases, disaggregases and proteases, which cooperate for the cellular proteostasis (Reinle et al. 2022). In this system, sHSP act first, by sequestering denaturing substrates. sHSP should form a stable complex with the denaturing client, until this can be either delivered to other chaperones of the system or targeted to degradation. When we tested the three sHSP for this holdase capacity, overall, we found a poor such ability. Only HSP1 and the mixture of all three sHSP contrasted CS precipitation. Indeed, though HSP2 and HSP3 could clearly delay CS aggregation, during early heat exposure, they did not seem capable of forming stable enough assemblies with it. HSP2 and HSP3 might have substrate preferences (Basha et al. 2004), or, simply, they do not work as holdases under the tested conditions. It is also possible that their physiological role in the cell does not imply the capacity to bind tightly to the denaturing substrate, which could be played by HSP1 only and/or by other chaperones. Some sHSP bind their clients stably, storing them for the disaggregating machineries, while others can associate only transiently with the substrate (de Miguel et al. 2009). Both sHSP types contribute to protein homeostasis and enhance bacterial cell survival under stress (Obuchowski et al. 2021). There are other examples of earlier investigated sHSP that are not effective in protecting heat-damaged substrates from insolubilisation (Basha et al. 2010; Obuchowski et al. 2019). Perhaps, in *L. plantarum*, a stable complex with unfolding substrates may require other partner proteins or more HSP types. Divergent functions, as well as functional cooperations, have been attributed to the sHSP from organisms possessing multiple members for this class of chaperones (de Miguel et al. 2009; Basha et al. 2010; Bepperling et al. 2012). Indeed, while most bacteria have a single sHSP, *E. coli* and other *Enterobacteriaceae* own two different sHSP, i.e., IbpA and IbpB, that have specialised towards different roles: the former acts as an efficient holdase, while the latter is a weak substrate-binder, but promotes the dissociation of assemblies, enhancing subsequent refolding of client substrates (Matuszewska et al. 2005; Ratajczak et al. 2009; Obuchowski et al. 2019). Very recently, Piróg et al. (2021) demonstrated that IbpA and IbpB do associate into a functional heterodimer, working as a two-protein sHSP machinery. Such cooperation might occur even for the three sHSP of *L. plantarum*. Based on our findings, it is tempting to speculate that HSP1, HSP2 and HSP3 could interplay for an efficient trapping of the substrate, possibly by forming mixed assemblies. However, this hypothesis needs further specific studies to be ascertained.

Fluorescence anisotropy measurement is a method of choice for studying changes in membrane fluidity. In the field of sHSP, few studies have focused on the regulation of membrane fluidity by these proteins, but rather on their physical interaction with the membrane. In this work, the fluidity of a model membrane reconstituted from lipids extracted from the plasma membrane of *L. plantarum* was monitored as a function of temperature variation. Thus, the potential lipochaperone activity of *L. plantarum* HSP1, HSP2 or HSP3 could be evaluated in vitro as previously described for other sHSP (Török et al. 2001; Coucheney et al. 2005; Maitre et al. 2014). Overall, at 30 °C, i.e., a physiological growth temperature for *L. plantarum*, all three sHSP determined a significantly different membrane fluidity, pointing to a potential interaction with liposomes. However, at heat stress temperature (i.e., 40–45 °C), only HSP2 would retain an anti-fluidising effect. Our results show a clear lipochaperone activity for HSP2, with a significant reduction in membrane fluidisation from 30 °C. These findings are similar to those obtained for other sHSP, which were ascribed a membrane-stabilising activity, such as Lo18 from *O. oeni*, HSP17 from *Synechocystis* or HSP16.3 from *Mycobacterium tuberculosis* (Török et al. 2001; Tsvetkovaet al. 2002; Coucheney et al. 2005; Zhang et al. 2005; Chowdary et al. 2007; Balogi et al. 2008; Maitre et al. 2014). Indeed, in the presence of these proteins, an improved physical order, in model lipid membranes, is observed when environmental conditions favouring membrane fluidisation are applied (i.e., heat, ethanol, benzyl alcohol in particular) (Török et al. 2001; Maitre et al. 2014). These different works suggest that sHSP-lipid associations are partly related to the change in membrane fluidity (which is temperature dependent) and partly linked to conformational changes of sHSP induced by the increase of temperature, allowing to expose certain regions of sHSP favourable to the interaction with the membrane (Zhang et al. 2005; Chowdary et al. 2007; Balogi et al. 2008; Maitre et al. 2014). For example, the electrostatic interactions keeping the proteins properly filled can be modified upon temperature increase, generating changes in their conformation and exposing their hydrophobic regions that could interact with membranes (Tsvetkovaet al. 2002; Chen et al. 2003). In the present work, no action of either sHSP was observed at temperatures below 30 °C, thus suggesting that a protein conformational change would be necessary for their interaction with the membrane. This in vitro study confirms the membrane fluidity regulation activity previously ascribed to HSP2, as earlier shown in vivo in *L. plantarum* (Capozzi et al. 2011b, a, 2012). Our data, therefore, reinforce the hypothesis that HSP2 has an important role due to its probable interaction with the membrane, allowing it to regulate its fluidity by a still unknown mechanism.

The addition of HSP3 resulted in a similar anisotropy profile as HSP2, by showing a modulation of membrane fluidity from 30 °C. However, this effect was no longer detected at 40 °C and even seemed to be reversed at 50 °C. Thus, the particular profile observed with HSP3 requires further investigation to understand how this protein functions in relation to the membrane as a function of temperature.

In summary, the data presented in this work establish the chaperone function of *L. plantarum* HSP1, HSP2 and HSP3. Their in vitro activity was found to be different in suppressing aggregation of thermally destabilised proteins, in the influence of pH conditions, in terms of holdase capacity and in the ability to modulate membrane fluidity. The specialization of *L. plantarum* sHSP may pertain even other functional aspects (e.g., modes of activation, client selection, etc.) that deserve further investigations. Moreover, future analyses shall be undertaken to understand the mechanism by which these three sHSP stabilise protein aggregates and interact with lipid bilayer. It is interesting to underline how this study contributes to proposing *L. plantarum* as one of the model bacteria for prokaryotes harbouring in their genomes three genes coding for sHSP.

## Supplementary Information

Below is the link to the electronic supplementary material.

Supplementary file1 (DOCX 292 KB)
