# Supplementary material for: Molecular chaperone function of three small heat-shock proteins from a model probiotic species
Source: Cell Stress Chaperones. 2022 Nov 22;28(1):79–89. doi: 10.1007/s12192-022-01309-6 (PMC9877261; doi:10.1007/s12192-022-01309-6)
Supplement: Supplementary file 1 — Supplementary file1 (DOCX 292 KB) [file 12192_2022_1309_MOESM1_ESM.docx]

**Supplementary figures**

**
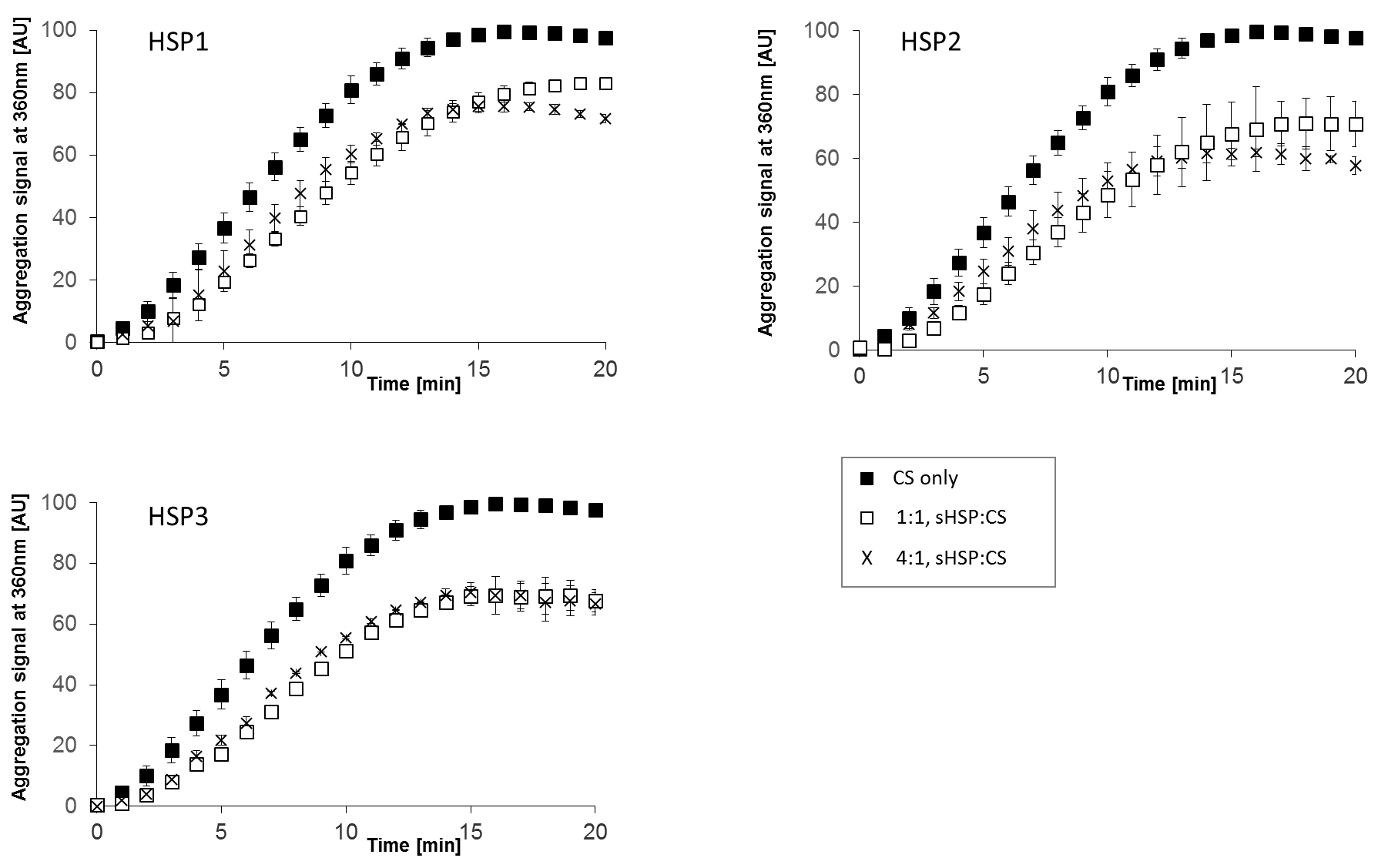
**

**Fig. S1**. **Inhibition of citrate synthase (CS) aggregation by *L. plantarum* sHSP.** CS (0.3 µM) was incubated at 45 °C without (solid square) or with HSP1, HSP2 or HSP3 at concentrations of 0.3 µM (molar ratio 1:1, sHSP:CS) (open square) or 1.2 µM (molar ratio 4:1, sHSP:CS) (cross). Protein aggregation was monitored by light scattering at 360 nm. The saturation signal was normalized to 100. Mean and SD from at least two experiments.


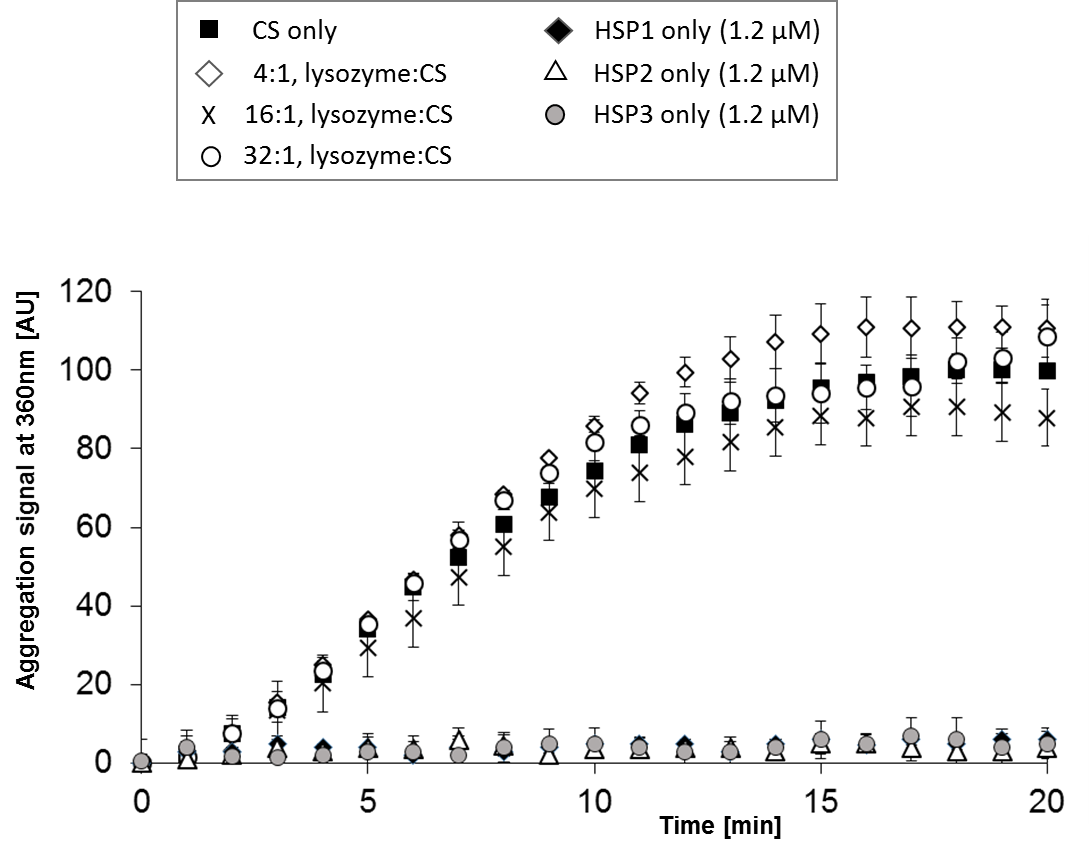


**Fig.S2. Aggregation signal of controls.** Aggregation was monitored as an increase of turbidity as measured at 360 nm. CS (0.3 µM) was incubated at 45 °C in absence (solid squares) or in presence of 1.2 µM (open diamond), 4.8 µM (cross), or 9.6 µM (open circle) lysozyme (i.e. molar ratio CS : lysozyme 1:4, 1:16, 1:32, respectively). Light scattering signal was also monitored in solution containing only 1.2 µM HSP1 (solid diamond), or HSP2 (open triangle) or HSP3 (gray circle). The saturation signal was normalized to 100. Mean and SD from at least two experiments.


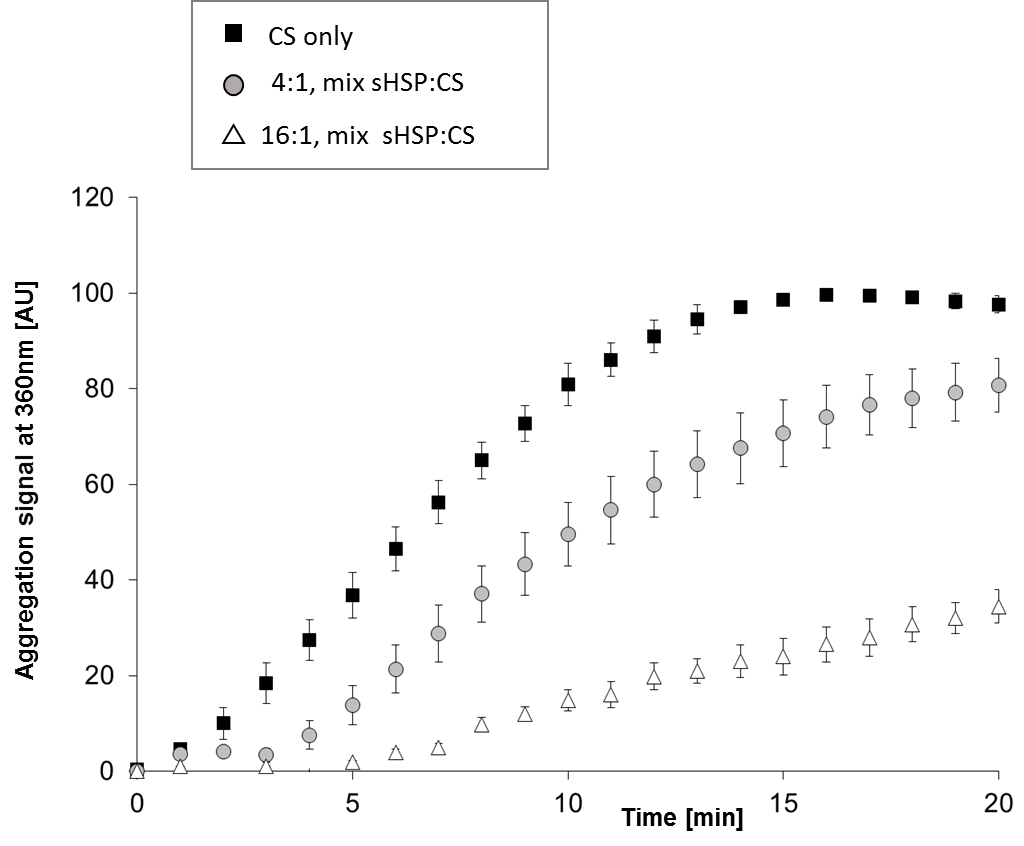


**Fig. S3. Aggregation of citrate synthase in the presence of a mixture of all three sHSP at pH 7.0.** CS (0.3 µM) was incubated at 45 °C, in Na-phosphate buffer pH 7.0, without (solid squares) or with HSP1 + HSP2 + HSP3, each at 0.4 µM (gray circle) or 1.6 µM (open triangle) so that their overall concentration would reach 1.2 µM or 4.8 µM, corresponding to a molar ratio of 4:1 or 16:1, sHSP : CS, respectively. The aggregation was monitored by the increase in light scattering at 360 nm. The saturation signal was normalized to 100. Mean and SD of at least two independent experiments.

**Supplementary tables**

**Table S1**. Bacterial strains, plasmids and oligonucleotides used in this study

| **Name** | **Description^a^ or nucleotide sequence^b^ (5’-3’)** | **Reference, source or application** |
| --- | --- | --- |
| **Bacterial strains** |  |  |
| *E. coli* MACH-1-T1 | DNA cloning host | Invitrogen (Life Technologies, Carlsbad, CA, USA) |
| *E. coli* BL21(DE3) | Recombinant protein expression | Invitrogen |
| 1 | Kan^r^; derivative of *E. coli* BL21(DE3) containing pET SUMO-HSP1 | This work |
| 16 | Kan^r^; derivative of *E. coli* BL21(DE3) containing pET SUMO-HSP2 | This work |
| 24 | Kan^r^; derivative of *E. coli* BL21(DE3) containing pET SUMO-HSP3 | This work |
| *L. plantarum* WCFS1 | Single-colony isolate of *L. plantarum* NCIMB8826; originally isolated from human saliva | (Kleerebezem et al. 2003) |
| **Plasmids** |  |  |
| pET SUMO vector | Kan^r^ vector allowing TA cloning of PCR products for *T7lac* promoter regulated expression in *E. coli* of N-terminally 6xHis- and SUMO-tagged proteins | Invitrogen |
| pET SUMO-HSP1 | pET SUMO derivative containing lp_0129 and encoding a recombinant HSP1, with an extra N-terminal region comprising a polyhistidine (6His) tag and the SUMO protein | This work |
| pET SUMO-HSP2 | pET SUMO derivative containing lp_2668 and encoding a recombinant HSP2, with an extra N-terminal region comprising a polyhistidine (6His) tag and the SUMO protein | This work |
| pET SUMO-HSP3 | pET SUMO derivative containing lp_3352 and encoding a recombinant HSP3, with an extra N-terminal region comprising a polyhistidine (6His) tag and the SUMO protein | This work |
| **Oligonucleotides** |  |  |
| Hsp1 FOR | ***ATG***GCTAATACTTTAATGAATCGGAAC | This work, *hsp1* (lp_0129) cloning |
| Hsp1 REV | ***TTA***TTGAATTTCGATTTGACCGTCATTC | This work, *hsp1* (lp_0129) cloning |
| Hsp2 FOR | ***ATG***CGCCAGCAATTATTCGGTCATC | This work, *hsp2* (lp_2668) cloning |
| Hsp2 REV | ***TTA***TTGTATTTCGATCTTACCTTCGCTATC | This work, *hsp2* (lp_2668) cloning |
| Hsp3 FOR | ***ATG***GCTAACGATATGATGAATTGGCAC | This work, *hsp3* (lp_3352) cloning |
| Hsp3 REV | CA***TTA***CTGAATTTCAATATGATGGGTATC | This work, *hsp3* (lp_3352) cloning |
| SUMO Forward | AGATTCTTGTACGACGGTATTAG | Invitrogen, sequencing |
| T7 Reverse | TAGTTATTGCTCAGCGGTGG | Invitrogen, sequencing |
| T7 promoter FOR | TTAATACGACTCACTATAGG | This study, PCR control on recombinant vectors |

^a^ Kan^r^, kanamycin resistant

^b^ start and stop codon sites are bold and italicized

**Table S2**. Biochemical parameters, deduced amino acid sequences and alignment of *L. plantarum* sHSP. Sequence alignment was carried out using Clustal Omega (<http://www.ebi.ac.uk/Tools/msa/clustalo/>); identical amino acid are highlighted in yellow; the α-crystallin domain, which folds into a β-sandwich with two anti-parallel sheets of three and four β-strands, was determined using Alpha-Fold (https://alphafold.ebi.ac.uk/) (Jumper et al. 2021; Varadi et al. 2022) and is in bold; within the C-terminus, the three-residue IX(I/V) motif, known to be involved in oligomerization (Delbecq S. P., Jehle S. 2012), is underlined.

|  | **chain length (aa)** | **Theoretical pI** | **Predicted Mw (kDa)** | **Observed Mw (kDa)** | **Molar extinction coefficient ε (M^-1^ cm^-1^)** |
| --- | --- | --- | --- | --- | --- |
| **HSP1** | 140 | 4.53 | 15.998 | 15.999 | 9,970 |
| **HSP2** | 139 | 5.81 | 16.082 | 16.084 | 9,970 |
| **HSP3** | 147 | 5.00 | 16.672 | 16.673 | 16,960 |

HSP2 MRQQLFGHH------LDDLLKPTKLLKRAHREAENVLNAHVGMK**TDVVEHDDDYTVTAEL**

HSP1 MANTLMNR--------NDFGMLDPFERMARSFWAPLENMDQVLK**TDINETDDQYQVKVDV**

HSP3 MANDMMNWHNDLFDRLNDWTKMDDLVNGFGRTFLNAGSHGSV**LKTDIKENDDQYTMKVDI**

* : :: :* : . . :***: * **:* :..::

HSP2 **PGFDKDAITVKYADEWLTIRAHRS--QDDRNDDGRVLHRERMDADFTRKFHLTNVVREEI**

HSP1  **PGIDKQDVKLDYRDNVLSIKVQKDSFVDHEDQDQNIVMNERHTGTLQRQYMLPNVAANKI**

HSP3  **PGIDKQNIALKYRDGTLSIAVKRDSISDESDKDGNIIASERQTGRFGRQYSLPDVDVDKI**

**:**: : :.* * *:* .::. *. :.* .:: ** . : *:: * :* ::*

HSP2 **QAHYQAGLLTVTLPKTV**AD-SEGKIEIQ

HSP1 **TASQADGVLTITLPK**TQPSANDGQIEIQ

HSP3 **EARYENGVLQLTLPKKA**AA-DTHHIEIQ

* *:* :****. . :****

**Identification of sHSP molecular weight by MALDI-TOF-MS**

A MALDI sample preparation protocol using MTP 384 target plate ground steel (Bruker Daltonics, Bremen, Germany) was employed. Purified sHSP were diluted in 1% acqueous solution of trifluoroacetic acid (TFA) and desalted using C18 ZipTips (Millipore, Watford, UK). One mL of this protein solution was mixed with an equal amount of matrix (20 mg α-cyano-4-hydroxycinnamic acid, HCCA, in 50% acetonitrile with 0.1% trifluoroacetic acid) and 0.5mL of the mixture were loaded directly onto a thin layer of HCCA crystals formed from 5 mg/ml solution of HCCA (5mg HCCA in acetonitrile/acetone 1/1). After ambient conditions drying, mass spectra of the protein spots were acquired on Autoflex III™ TOF/TOF200 instrument (Bruker Daltonics) with smartbeamTM laser technology. All spectra were acquired in the positive reflecting mode with 200 Hz laser frequency, in the 4000-20000m/z range. MS spectra were externally calibrated using abundant fragment ion peaks derived from Protein Calibration Standard I (Bruker Daltonics) (including insulin, ubiquitin, myoglobin, and cytochrome c [M+H]+ and [M+2H]2+ ions, as well as the protein dimers).

**References**

Delbecq S. P., Jehle S. KR (2012) Binding Determinants of the Small Heat Shock Protein, αB-crystallin: Recognition of the “IxI” Motif. EMBO J 31:4587–4594. https://doi.org/10.1038/emboj.2012.318

Jumper J, Evans R, Pritzel A, et al (2021) Highly accurate protein structure prediction with AlphaFold. Nature 596:583–589. https://doi.org/10.1038/s41586-021-03819-2

Kleerebezem M, Boekhorst J, Van Kranenburg R, et al (2003) Complete genome sequence of Lactobacillus plantarum WCFS1. Proc Natl Acad Sci U S A 100:1990–1995. https://doi.org/10.1073/pnas.0337704100

Varadi M, Anyango S, Deshpande M, et al (2022) AlphaFold Protein Structure Database: Massively expanding the structural coverage of protein-sequence space with high-accuracy models. Nucleic Acids Res 50:D439–D444. https://doi.org/10.1093/nar/gkab1061
